# Mindfulness may be associated with less prosocial engagement among high intelligence individuals

**DOI:** 10.1038/s41598-023-31039-3

**Published:** 2023-03-14

**Authors:** Qingke Guo, Sisi Li, Jingu Liang, Xinxin Yu, Yiqing Lv

**Affiliations:** 1 Department of Psychology, Guangxi Normal University, Guilin, 541004 China; 2 Department of Psychology, Shandong Normal University, Jinan, 250014 China

**Keywords:** Psychology, Human behaviour

## Abstract

This study examined the role of dispositional mindfulness in the association between intelligence and prosocial behavior. A total of 759 college students (mean age is 22.03; 477 females) participated in exchange for extra credit in psychology course. The results confirmed a positive relationship between intelligence and prosocial behavior as revealed by many studies, with empathy serving as a potential mediator. Mindfulness negatively moderated all the hypothesized pathways between research variables. Specifically, with the increase of the levels of dispositional mindfulness, (1) the intelligence-prosociality association changed from positive to negative, (2) the intelligence-empathy association changed from positively significant to insignificant, (3) the empathy-prosociality association changed from stronger to weaker. These findings may suggest some limitations of mindfulness. That is, present moment awareness and acceptance of the status quo may result in reduced arousal when witnessing others suffering, thereby preventing high intelligence individuals from helping the sufferers to get rid of trouble.

## Introduction

Prosocial behavior refers to voluntary actions aiming to benefit others^1^, such as helping, sharing, cooperating, donating, caring, and comforting. Prosocial behavior requires accurate perception and understanding of the desires of the victim, and proper decisions that meet the need of the victim. In the process of prosocial engagement, theory of mind abilities and general cognitive abilities are greatly needed^2–5^. The above reasoning suggests that high intelligence individuals are more likely to be prosocial, which has been supported by many studies. For example, verbal ability is found to be a good predictor of the participation of charitable giving and the amount of donation, even after controlling for income, wealth, education, subjective health, and personality^6^. High intelligence individuals can be more generous in economic games^7^. They tend to trust others, and thus are more likely to engage in prosocial actions^8^. Aranda and Siyaranamual (2014) found that mathematical and verbal abilities were both positively associated with civic engagement (e.g., doing voluntary and charity work, engaging in political or community-related activities^9^). A meta-analysis of the repeated prisoner's dilemma game at multiple colleges showed that every 100-point increase in a college's average SAT score (a proxy for cognitive abilities^5^) averagely result in an improvement of the students' cooperation rate by 5–8% in that college^10^. Millet and Dewitte argued that altruistic behavior can be considered by the participants as a costly signal of fitness. Altruistic behavior can convey desirable traits that cannot be directly observed, such as social status, generosity, kindness, and trustworthiness^7^. Highly intelligence individuals are better in realizing the long-term benefits of prosocial behavior, which may include good social prestige, more opportunities to be selected as a partner or mate. Therefore we propose Hypothesis 1: There is a positive correlation between intelligence and prosocial behavior.

Empathy is an important driving force of prosocial engagement^11^. Witnessing the misfortune of victims elicit emotional responses such as pity and sympathy in the witness, which prompts helping behaviors to relieve suffering of the victims^12^. Individuals with stronger theory of mind and perspective taking abilities are better in understanding the victim's thoughts and feelings and put themselves in the victim's position, and therefore tend to lend a helping hand^4^. Empirical research shows that high intelligence individuals are sensitive to the thoughts and feelings of others, and are easier in generating other-centered feelings^3^. The above arguments suggest that empathy may potentially serve as a mediating mechanism in the relationship between intelligence and prosocial behavior. Therefore we propose Hypothesis 2: the intelligence-prosociality association may be mediated by empathy.

Mindfulness not only enhances well-being of the self, but also benefits others^13^. Meta-analysis studies found that mindfulness is positively associated with prosocial behavior, regardless mindfulness was operated as a personal disposition, a state induced by experimental manipulation, or an ability enhanced by training^13^. One reason is that mindfulness increases moral awareness or sensitivity to morally relevant information^14–16^. Featured by openness and unbiased awareness, a mindful state can enhance sensitivity to morally relevant internal and external cues^14^. Mindfulness entails sustained attention, increasing the ability to be aware of the needs of others in social environments. Another reason is that mindfulness is associated with improved emotion regulation ability, which can result in more prosocial engagement, especially in situations that making a prosoical decision involves negative emotions^13^. Furthermore, mindfulness facilitates more empathic responses by reducing self-referential thoughts and emotions, boosting the motivation to help the suffering others. Evidence shows that mindfulness trainees are more likely to help an ostracized stranger and socially include her/him in interpersonal interactions^17^.

Previous research has suggested that mindfulness can interact with other important personal dispositions to influence psychosocial outcomes. For example, high (relative to low) dispositional mindfulness individuals can experience greater loss of self-control after performing surface acting^18^. Also there is evidence showing that mindfulness and self-construal interact to influence prosocial behavior^19^. Specifically, people with more independent self-construal (and those who were experimentally manipulated to have a more independent self-construal) were less helpful after mindfulness training than controls. Another recent study found that mindfulness intervention has a stronger effect on prosocial engagement among individuals with higher levels of moral identity (a moral disposition positively associated with intelligence^3^), suggesting that moral awareness or moral sensitivity is more likely to be enhanced by mindfulness training among highly ethical individuals^16^. This indicates that mindfulness and intelligence may interact to influence prosocial behavior. People with high intelligence can process environmental information more effectively. Therefore we propose that the association between intelligence and moral awareness can be enhanced when self-referential thoughts/emotions are reduced and consequently empathic responses are boosted. That is, mindfulness can enhance sensitive to moral issues and prosocial engagement^20^, but this positive effect is suggested to be greater among high (relative to low) intelligence individuals. Thus we propose Hypothesis 3: the relationship between intelligence and prosocial behavior can be enhanced by mindfulness.

**Figure 1:**
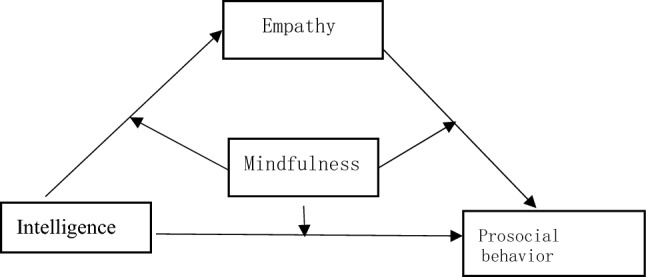
Diagram of the hypothesized model.

There is no evidence in the existing literature on how the relationship between intelligence and empathy as well as between empathy and prosocial behavior can be moderated by mindfulness. In this study we tentatively constructed a moderated mediation model^21^ to make an exploration (Fig. 1). Specifically, we assume that the relationships between intelligence and prosocial behavior, intelligence and empathy, and empathy and prosocial behavior may all be moderated by mindfulness.

## Methods

### Participants

Questionnaires were administrated to students who enrolled in a psychology course. They participated in order to earn extra credit. Informed consent was obtained. The participants were told that their response to questionnaire items would be used exclusively in a research project and kept confidentially. After excluding cases with invalid or incomplete responses, totally 759 undergraduate students (N_female_ = 477, M age = 22.03, SD = 1.63) were included in the research sample. Eight-three percent of the students are atheists, and the percentage of students born in cities, towns, and rural areas were 16.3%, 38.6%, and 56.8%, respectively. Most of them have a monthly family income of about 3500 CNY. In the treatment of human participants, this study complies with the American Psychological Association ethical standards and the 1964 Helsinki declaration and its later amendments or comparable ethical standards, and was approved by the academic committee at Guangxi Normal University.

### Measures

#### Self-reported prosocial behavior

The Self-Report Altruism Scale Distinguished by the Recipient (SRAS-DR) was developed by Oda et al.^22^. SRAS-DR consists of 21 items measuring three dimensions: altruism to kin, altruism to friends, and altruism to strangers. SRAS-DR has showed good reliability and validity in Chinese populations^3^. Example items are "I have listened to the complaints of friends or acquaintances", "I have supported family members when they are feeling down". A 5-point scale was used, with 1 indicating complete disagreement and 5 indicating complete agreement. The total score was taken, with higher scores indicating higher levels of prosocial behavior. In this study, Cronbach's alpha of the whole scale was 0.96, Cronbach's alpha of three subscales were 0.91 (altruism to kin), 0.93 (altruism to friends), and 0.88 (altruism to strangers).

#### Intelligence

The Chinese version^3^ of the Raven's Standard Progressive Matrices (SPM^4,23^) was used to measure fluid intelligence. SPM items were divided into 5 parts (A, B, C, D, and E), with each part containing 12 items with gradually increasing difficulty. When responding to each item, participants were asked to find the correct answer from 6 or 8 options and fill it in the missing part of the geometric figure. The possible score for each item is 1 (correct) or 0 (false). The total score was used to represent the level of fluid intelligence. In this study, only the 30 difficult items (the last 6 questions in each part) were used. Cronbach's alpha was 0.89 in this study.

#### Empathy

The Chinese version of the Interpersonal Reactivity Indicator Scale^24^ (Davis, 1983) was used (C-IRI^3^). C-IRI contains 22 items, measuring four dimensions of empathy, namely perspective taking, fantasy, empathic concern, personal distress. Perspective taking measures the ability to recognize and appreciate the perspectives of others; fantasy measures empathic responses to characters in movies, novels, plays, and other fictional situations; empathic concern measures other-oriented emotions (e.g., tenderness, sympathy, compassion) in response to the person in need; personal distress measures self-oriented negative emotional responses (e.g., anxiety, uneasiness) when witnessing other people’s distress. Perspective taking and fantasy reflect cognitive responses; while empathic concern and personal distress reflect emotional sharing competence^25^. The four components of empathy showed different function in predicting prosocial behavior^24,25^, but only the total score was used in this study for conciseness. Cronbach's alpha of the whole scale was 0.82, Cronbach's alpha of the four subscales were 0.77 (perspective taking), 0.59 (fantasy), 0.66 (empathic concern), and 0.78 (personal distress), respectively.

#### Dispositional mindfulness

Dispositional mindfulness was measured using the Chinese version of the Mindful Attention Awareness Scale (MAAS^26^). The scale includes 15 items, such as "When a bad mood occurs, you should not avoid it, but let it go away slowly", "I always pay attention to my physical feeling and mental state". The participants are asked to respond to each item on a 6-point scale (1 = almost never, 6 = almost always) according to their own experiences. A total score was taken, with higher scores indicating higher levels of mindfulness. In this study Cronbach's alpha was 0.77.

## Results

SPSS (version 20) was used for descriptive statistical analysis of the data, Model 4 of the PROCESS micro (version 4.1) for SPSS was employed for mediation analysis, and Model 59 was employed for moderated mediation analysis^21^. Hypothetical models were tested by estimating 95% confidence intervals (CIs) for mediation and moderation effects using 5000 bootstrap samples. The results we reported were that without controls (e.g., sex, parental education) in our equations, which were similar to that with controls.

### Correlations between intelligence, prosocial behavior, empathy, and mindfulness

Descriptive statistics and correlations of key variables are presented in Table 1. The results showed that intelligence, empathy, prosocial behavior, and mindfulness are significantly and positively correlated with each other. Mindfulness and other variables are also correlated, which confirmed the positive association between mindfulness and empathy^25^. But the correlation coefficients are not very large, suggesting moderation analyses can be conducted^21^_._

**Table 1:** Descriptive statistics and correlation analysis (n = 759).

	M	SD	1	2	3	4
1. Intelligence	17.10	6.14	0.89			
2. Empathy	72.45	11.63	0.30**	0.82		
3. Prosocial behavior	82.57	16.03	0.29**	0.34**	0.96	
4. Mindfulness	56.85	7.62	0.28**	0.21**	0.38**	0.77

**p < 0.01.

### Mediating effect of empathy and the moderating effect of mindfulness

#### Mediation analysis

Mediation analysis regarding the role of empathy is conducted (Tables 2, 3). In Equation (1), intelligence (beta = 0.29) positively predicted prosocial behavior; in Equation (2), intelligence (beta = 0.30) positively predicted empathy; in Equation (3), intelligence (beta = 0.20) and empathy (beta = 0.28) both positively predicted prosocial behavior (Table 2). As a result, the 95% Bootstrap CIs for the direct and indirect effects of intelligence on prosocial behavior did not contain 0. The relationship of intelligence and prosocial behavior was partially mediated by empathy, accounting for more than a quarter of the total effect (Table 3).

**Table 2:** Mediating effect of empathy in intelligence-prosociality association.

Predictors	Equation (1): Prosocial behavior			Equation (2): Empathy			Equation (3): Prosocial behavior		
	*B*	Bootstrap SE	t	*B*	Bootstrap SE	t	*B*	Bootstrap SE	t
Intelligence	0.75	0.091	8.26***	0.56	0.066	8.51***	0.53	0.09	5.85***
Empathy							0.39	0.05	7.99***
R^2^	0.083			0.087			0.154		
F	68.18***			72.45***			68.80***		

***p < 0.001.

**Table 3:** Bootstrap analysis of mediation effects.

Effect type	Effect	Bootstrap SE	% of total effect	Bootstrap 95% CI	
				Lower limit	Upper limit
Total effect	0.75	0.09		0.51	0.93
Direct effects	0.53	0.09	71.25%	0.35	0.71
Indirect effects	0.22	0.05	28.75%	0.13	0.31

#### Moderated mediation analysis

We further tested whether the effect of intelligence on empathy and prosocial behavior, and the effect of empathy on prosocial behavior are moderated by mindfulness (Table 4). In Eq. (1), the product term of mindfulness and intelligence has a significance influence on empathy, indicating that mindfulness plays a moderating role in the intelligence-empathy association. In Eq. (2), the product term of empathy and mindfulness, and the product term of intelligence and mindfulness both have a significance influence on prosocial behavior, indicating that mindfulness can play a moderating role in the association of empathy and prosocial behavior, and the association of intelligence and prosocial behavior.

**Table 4:** The moderating effect of mindfulness.

Variable	Equation (1): Empathy			Equation (2): Prosocial behavior		
	*B*	SE	t	*B*	SE	t
Intelligence	2.73	0.46	5.92***	4.07	0.65	6.30***
Empathy				1.04	0.29	3.61***
Mindfulness	0.93	0.16	5.94***	2.79	0.35	8.09***
Intelligence × mindfulness	− 0.04	0.01	− 4.91***	− 0.07	0.01	− 5.91***
Empathy × mindfulness				− 0.014	0.005	− 2.70**
R square	0.13			0.29		
F	38.31***			61.53***		

**p < 0.01, ***p < 0.001.

Simple slopes analysis is conducted to elaborate the moderating effect of mindfulness. Specifically, slopes are computed and compared when mindfulness is high (1 standard deviation above the mean) and when mindfulness is low (1 standard deviation below the mean).

**Figure 2:**
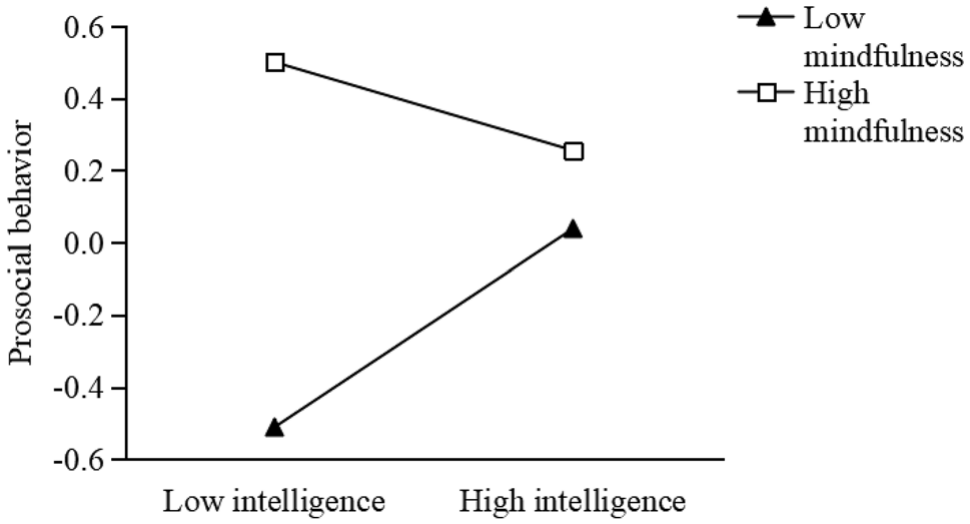
Mindfulness moderates the relationship between intelligence and prosocial behavior.

First, we use mindfulness as a moderator in the intelligence-prosociality association (Fig. 2). The results show that intelligence is positively associated with prosocial behavior (B = 0.67, t = 6.16, p < 0.001) when mindfulness is low. However, when mindfulness is high the intelligence-prosociality association turn out to be negative (B = − 0.29, t = − 2.20, p < 0.001). This suggests that the intelligence- prosociality association can be weakened by mindfulness.

**Figure 3:**
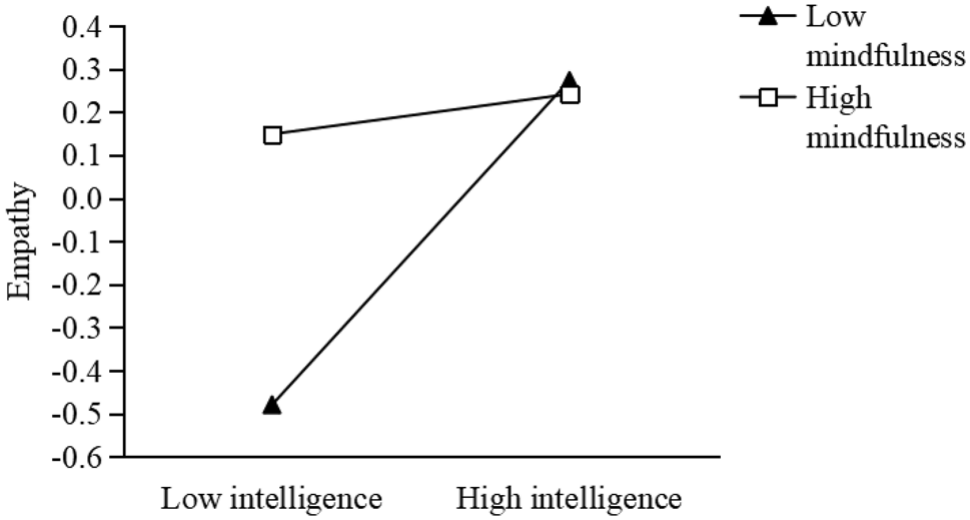
Mindfulness moderates the relationship between intelligence and empathy.

Second, we use mindfulness as a moderator in the intelligence-empathy association (Fig. 3). The results show that intelligence can positively predicts empathy at lower levels of mindfulness (B = 0.68, t = 8.78, p < 0.001); when mindfulness is high, the intelligence-empathy association turn out to be insignificant (B = 0.11, t = 1.05, p > 0.05).

**Figure 4:**
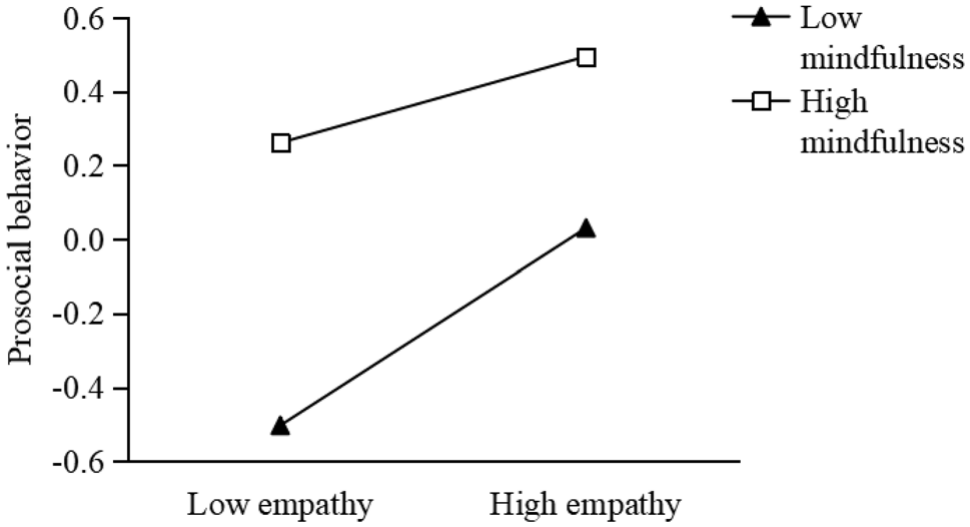
Mindfulness moderates the relationship between empathy and prosocial behavior.

Third, we use mindfulness as a moderator in the empathy- prosociality association (Fig. 4). The results show that though empathy is positively associated with prosocial behavior regardless of the mindfulness levels of the participants, the association is stronger among participants with low (B = 0.36, t = 6.42, p < 0.001) relative to high (B = 0.17, t = 2.83, p < 0.01) dispositional mindfulness. This suggests that mindfulness may reduce the link between prosocial emotions and behavior.

The above simple slopes analyses suggest that the mindfulness may prevent high intelligence individuals from generating prosocial emotions and behavior.

## Discussion

This study intends to confirm the relationship between intelligence and prosocial behavior and the potential mediating role of empathy. More importantly, we introduce mindfulness as a moderator to explore how mindfulness interacts with intelligence to influence prosocial emotions and behavior.

### Intelligence and prosocial behavior mediated by empathy

Consistent with Hypothesis 1, we found a positive correlation between intelligence and prosocial behavior. This is in line with many previous studies^3,7,9^ which have afforded several explanations. First, altruism may serve as a coping strategy to enhance overall fitness. Altruistic behavior signals trustworthiness, which helps the actors gain more coalition partners and potential mates^6,27^. Second, high intelligence individuals are more aware of the long-term benefits of prosocial behavior^3^. They realize that a good reputation is associated with better access to resources and can enhance overall fitness in the long run^28^. Therefore they are less likely to engage in unethical behavior that may damage their reputation^29^. Third, high intelligence individuals have more resources thus prosocial behavior is relatively less costly for them. And they are more competent in regaining the sacrificed resources^30^. Fourth, high intelligence individuals are competent in perceiving and recognizing the needs of the victims and coming up with effective ways to help the victims^30^.

Consistent with Hypothesis 2, this study found that empathy may be a candidate mediator in the intelligence- prosociality association. This confirms previous findings that high intelligence individuals are more sensitive to the thoughts and feelings of others, and are more likely to have other-centered feelings^3^. High intelligence individuals have stronger executive function and theory of mind abilities, therefore it easier for them to put themselves in other people's situations and lend a helping hand when witnessing others suffering.

### The negative moderating effect of mindfulness

This study found that mindfulness moderates the direct pathway between intelligence and prosocial behavior, but the direction is contradictory to Hypothesis 3. We found that the intelligence and prosocial behavior is positive in low mindfulness condition and negatively in high mindfulness condition (Fig. 1). Recent studies suggest that mindfulness does not enhance prosocial behavior in all people. For example, a study finds that for those with a dependent self-construal, experimentally induced mindfulness increases prosocial behavior. However, for those with an independent self-construal, mindfulness reduced prosocial behavior^19^. A possible explanation is that mindfulness facilitates people to focus attention on the present rather than the future, and thus result in reduced arousal and lower motivation to take actions^31^. Mindfulness entails non-judgment and non-reactivity to inner and outer experiences, which may lead to neutral and emotionless responses to the needs of others^32^. Another explanation is that emotional regulation abilities cultivated by mindfulness practice may also undermine prosocial engagement in some situations. Negative emotions such as guilt are important drivers of prosocial behavior^31^. Many helpful behaviors are implemented in order to eliminate the helper’s own negative emotions, such as personal distress and guilt^12,31^. Mindfulness can enhance awareness or sensitivity to moral issues^14–16^, but this may not be true in some occasions, or for all individuals.

We also found that the relationship between intelligence and empathy declines with increasing levels of mindfulness (Fig. 2). That is, the intelligence-empathy association is positively significant when mindfulness is low, and is insignificant when mindfulness is high. This is consistent with the findings that focused breathing mindfulness practice leads to reduced future focus and thus low arousal, hindering the generation of prosocial emotions (e.g., guilt^31^).

Furthermore, mindfulness significantly reduced the association between empathy and prosocial behavior. Empathy is positively associated with prosocial behavior among participants with both high and low dispositional mindfulness (Fig. 3), but the strength of association is significantly weakened as the level of mindfulness increased. This is consistent with the findings that mindfulness attenuates behavioral responses to external cues^33^. This can also be explained by the fact that mindfulness reduces the motivation to take action to change the status quo^34^, because such actions may interfere with their peaceful and relaxed state. Mindfulness favors focused attention on the present moment and acceptance of the status quo, thereby preventing people from taking action to reach a desired state. In social situations when seeing a victim suffering the witness will automatically generate empathic responses (e.g., empathic concern, personal distress). The desired state is that the victim being get rid of trouble otherwise the witness will experience negative emotions such as guilt, remorse, and distress^1,12^. The above reasoning suggests that some features of mindfulness (e.g., reduced future focus and acceptance of the status quo) may to some extent prevent prosocial emotions turning into prosocial behavior^34^.

In recent years, some scholars have begun to pay attention to the limitations of mindfulness^35–37^. Focused breathing mindfulness does not promote psycho-social development under all conditions, and bring benefit to everyone^19^. For example, mindfulness training can lead to false memories^38^. Mindfulness reduces people's prosocial reparatory behaviors after committing an ethical transgression^32^. These limitations may be overcome when loving kindness mediation that cultivates other focused emotions is practiced^31^. Findings of this study suggest that mindfulness may reduce prosocial engagement among high intelligence individuals, and it may further weaken the association between intelligence and empathy, and the association between empathy and prosocial behavior. This is consistent with the findings that mindfulness may prevent the generation of prosocial emotions, and undermine the influence of prosocial emotions on prosocial behavior^31^. This may be especially true for high intelligence individuals because taking actions that may interfere with the peaceful and relaxed state to change the status quo can be more costly (Supplementary Information).

## Limitations and future directions

Several limitations have to be addressed. First, the self-report measure (i.e., the Mindful Attention Awareness Scale) used in this study may not be adequate in capturing Buddhist conceptions of mindfulness. This scale mainly assesses individual difference in attention to and awareness of the present experiences, ignoring other dimensions of mindfulness (e.g., acceptance, non-judgment, non-reactivity) that influence psycho-social functioning^39^. Second, the use of self-report measure may introduce social desirability and other response biases that can ruin relationships among research variables^39^. Previous studies show that high mindfulness individuals tend to act in an honest, modest, and harmless way, suggesting that they make less socially desirable responding^40,41^. This may be one reason why mindfulness has a negative moderating effect in the intelligence-prosociality association. But this problem has not been solved (e.g., by using social desirability as a statistical control) in this study. Third, there may be a ceiling effect when gathering data using a prosocial measure that comprises items having only five options^42^. Fourth, failing to control individual-level confounding variables such as self-construal^19^, moral dispositions^16^, and demographic factors is another limitation of this study. These variables may influence the relationship between intelligence and prosocial behavior. Fifth, intelligence and mindfulness both contribute to prosocial emotions and behaviors, leading to the fact that the effect of one variable interfered by the other. Sixth, participants of this study are exclusively Chinese. Chinese society has been greatly modernized in recent years, suggesting that the psychological difference between residents in China and other parts of the world is becoming smaller^43,44^. However, this does not mean that findings of this study can be generalized to other cultures. Seventh, a cross-sectional design limits this study’s power to make causal inference. Future studies are encouraged to operationalize mindfulness in laboratory settings to engender more sound findings. Finally, our sample size may not be large enough to achieve sufficient power to detect interactions^45^.

## Conclusion

This study finds that intelligence is positively associated with prosocial behavior via empathy, providing more evidence on the role of cognitive ability in psychosocial development. This study may have revealed the limitations of mindfulness. That is, the intelligence-prosociality association is weakened by mindfulness, suggesting that mindfulness may deter prosocial engagement among high intelligence individuals. Furthermore, the intelligence-empathy association and the empathy-prosociality association can also be weakened by mindfulness. The reason may be that present moment awareness and acceptance of the status quo can result in reduced arousal thereby preventing high intelligence individuals from taking action to reach a desired state (e.g., help a victim get rid of trouble). In other words, high intelligence individuals are more likely to reach a peaceful and relaxed state by focusing on the present moment, thus they are reluctant to take action to reach a desired state. Though has several limitations, this study may be practically important in revealing drawbacks of traditional mindfulness practice.

## Supplementary Information

Supplementary Tables.

## Data Availability

The raw data that support the findings of this study are publicly available from the corresponding author.
