# Supplementary material for: Mindfulness may be associated with less prosocial engagement among high intelligence individuals
Source: Sci Rep. 2023 Mar 14;13:4208. doi: 10.1038/s41598-023-31039-3 (PMC10015037; doi:10.1038/s41598-023-31039-3)
Supplement: Supplementary file 1 — Supplementary Tables. [file 41598_2023_31039_MOESM1_ESM.docx]

Supplemental material

The scores of empathic concern and perspective taking were summed into an indicator of empathy. Mediation analysis (Table s1) and moderated mediation (Table s3) analysis were conducted using this indicator instead of the total score of IRI. Furthermore, the scores of fantasy and personal distress were also summed into another indicator of empathy (Table s2 and s4). The results remain unchanged. This justified previous proposition that different dimensions of empathy function as a whole, at least in some situations.

Table s1. Mediating effect of empathy1 in intelligence- prosociality association

| Predictors | Equation 1: Prosocial behavior | | | Equation 2: Empathy1 | | | Equation 3: Prosocial behavior | | |
| --- | --- | --- | --- | --- | --- | --- | --- | --- | --- |
|  | *B* | bootstrap SE | t | *B* | bootstrap SE | t | *B* | bootstrap SE | t |
| Intelligence | 0.75 | 0.091 | 8.26^***^ | 0.36 | 0.038 | 9.70^***^ | 0.44 | 0.09 | 4.76^***^ |
| Empathy1 |  |  |  |  |  |  | 0.85 | 0.08 | 10.39^***^ |
| R^2^ | 0.083 | | | 0.11 | | | 0.20 | | |
| F | 68.18^***^ | | | 94.04^***^ | | | 92.88^***^ | | |

Note: Empathy1 = perspective taking + empathic concern; ^***^*p*<0.001

Table s2. Mediating effect of empathy2 in intelligence- prosociality association

| Predictors | Equation 87rosocial behavior | | | Equation 2: Empathy2 | | | Equation 3: Prosocial behavior | | |
| --- | --- | --- | --- | --- | --- | --- | --- | --- | --- |
|  | *B* | bootstrap SE | t | *B* | bootstrap SE | t | *B* | bootstrap SE | t |
| Intelligence | 0.75 | 0.091 | 8.26^***^ | 0.19 | 0.039 | 5.02^***^ | 0.69 | 0.09 | 7.54^***^ |
| Empathy2 |  |  |  |  |  |  | 0.30 | 0.08 | 3.60^***^ |
| R^2^ | 0.083 | | | 0.032 | | | 0.10 | | |
| F | 68.18^***^ | | | 25.20^***^ | | | 41.10^***^ | | |

Note: Empathy2 = fantasy + personal distress; ^***^*p*<0.001

Table s3. The moderating effect of mindfulness

| Variable | **Equation 1:** Empathy1 | | | Equation 2: Prosocial behavior | | |
| --- | --- | --- | --- | --- | --- | --- |
|  | *B* | SE | t | *B* | SE | t |
| Intelligence | 1.25 | 0.33 | 4.70^***^ | 4.09 | 0.65 | 6.29^***^ |
| Empathy1 |  |  |  | 1.62 | 0.45 | 3.63^***^ |
| Mindfulness | 0.58 | 0.09 | 6.66^***^ | 2.39 | 0.28 | 8.63^***^ |
| Intelligence × mindfulness | -0.02 | 0.005 | -3.86^***^ | -0.07 | 0.01 | -5.92^***^ |
| Empathy1 × mindfulness | |  |  | -0.02 | 0.01 | -2.34^**^ |
| R square | 0.21 | | | 0.30 | | |
| F | 65.81^***^ | | | 65.89^***^ | | |

Note: Empathy1 = perspective taking + empathic concern; ^**^*p*<0.01, ^***^*p*<0.001

Table s4. The moderating effect of mindfulness

| Variable | **Equation 1:** Empathy2 | | | Equation 2: Prosocial behavior | | |
| --- | --- | --- | --- | --- | --- | --- |
|  | *B* | SE | t | *B* | SE | t |
| Intelligence | 1.48 | 0.27 | 5.40^***^ | 4.79 | 0.61 | 7.83^***^ |
| Empathy2 |  |  |  | 1.62 | 0.53 | 3.07^**^ |
| Mindfulness | 0.35 | 0.09 | 3.78^***^ | 2.94 | 0.35 | 8.46^***^ |
| Intelligence × mindfulness | -0.02 | 0.005 | -4.67^***^ | -0.08 | 0.01 | -7.27^***^ |
| Empathy2 × mindfulness | |  |  | -0.02 | 0.01 | -2.63^**^ |
| R square | 0.06 | | | 0.27 | | |
| F | 16.92^***^ | | | 54.72^***^ | | |

Note: Empathy1 = fantasy + personal distress; ^**^*p*<0.01, ^***^*p*<0.001
